# Special Issue “Exosomes and Extracellular Vesicles in Health and Diseases 2.0”

**DOI:** 10.3390/ijms252212447

**Published:** 2024-11-20

**Authors:** Francesca Beretti, Manuela Zavatti

**Affiliations:** Department of Biomedical, Metabolic and Neural Sciences, University of Modena and Reggio Emilia, Via del Pozzo 71, 41125 Modena, Italy

All cells secrete various types of membrane vesicles, known as extracellular vesicles (EVs), and this process is conserved throughout evolution. The secretion of EVs, initially described as a means of removing unnecessary compounds from the cell, is now recognized as a method of communication with distant cells [1].

An increasing number of studies suggests that EVs display considerable heterogeneity in terms of their size, distinct biological characteristics and complex formation mechanisms. The current classification system primarily distinguishes EVs into three key types:

-Exosomes, ranging in diameter from 30 to 150 nm, are formed through the fusion of multivesicular bodies with the cell membrane;

-Microvesicles, sometimes called ectosomes, which have diameters between 100 and 1000 nm, are generated through the direct budding of the cell membrane;

-Apoptotic bodies, encapsulating cytoplasm and organelles within a membrane, are created during cellular apoptosis and measure between 800 and 1000 nm in diameter [2].

Considering that EVs play roles in exchanging components between cells, the release mechanisms of protein and various nucleic acid fragments, and the expulsion of obsolete cell and membrane constituents, the characterization of EVs offers a promising non-invasive approach for screening biological samples from patients with different pathological and physiological states and has the potential to contribute to improving prognoses and/or diagnoses [3,4,5]. For example, EVs derived from cancer cells offer new insights and biomarkers for the diagnosis and prognosis of the most common diseases [6]. Undoubtedly, there are limitations surrounding vesicle isolation and determining whether isolated EVs are viable candidates for clinical use [7]; when considering EVs as biomarkers, two critical factors must be considered: the effective isolation of EVs and the selection of an appropriate analysis method [8].

EVs also have therapeutic potential, as they are important mediators in physiological and pathological processes. There has been growing interest in EVs for medical applications, and their roles in cell–cell communication indicate their relevance in biomedicine. EVs are known to protect their cargo, making them attractive carriers for targeted drug transport [9]. These drug delivery properties are intrinsic because of their lipid composition and protein content, which can influence their affinity to specific body regions. In this context, EVs can be loaded with various therapeutic agents and used as carriers for anti-tumor therapies. Their natural ability to target specific cell populations makes them an ideal medium for precise drug delivery. Furthermore, EVs are exciting nanocarriers candidates to treat brain disorders, as they may be able to cross the blood–brain barrier.

Researchers have focused on developing smart drug delivery systems using EVs with better targeting, safety, and pharmacokinetics than synthetic nanocarriers [10]. Since they are less dangerous than synthetic drugs, EVs are preferred as medicinal carriers. Moreover, they have a low immunological response, making them suitable for blood and plasma transfusions [6].

Taking the above into account, the aim of the second edition of this Special Issue was to collect both review and original research articles that investigate and elucidate the role of extracellular vesicles (EVs) in determining a patient’s physiological and pathological state in order to provide support for the development of potential novel diagnostic and therapeutic approaches. Through a rigorous peer-review process, ten articles were accepted, including six original articles, three review articles and one opinion article.

Given the considerable and growing number of existing works focusing on Matrix-Assisted Laser Desorption Ionization (MALDI)/Mass Spectrometry (MS), which provides valuable insights into the proteomic profiles of EVs, enabling researchers to propose potential biomarkers for disease diagnosis and prognosis, the work of Aresta et al. (contribution 1) comprehensively reviews the related literature. In particular, it is evident that the standardization of isolation protocols, integrating analytical methods into clinical workflows, and addressing the heterogeneity of the EV population have been shown to be crucial for EV characterization.

The report of Weber et al. (contribution 2) isolates and characterizes salivary EVs from COVID-19-positive subjects and their in-house negative healthy close contacts and explore their proteomic cargo using a mass spectrometry-based proteomics approach. Notwithstanding the small sample size and the necessity of functional validation experiments, the enriched terms of salivary EV proteomic cargo in the COVID-19 group suggest an association with immune response processes, oxygen transport, and antioxidant mechanisms. In contrast, in the healthy close contacts group, the enriched terms of the salivary EV signature profiles suggest processes related to epithelial homeostasis.

The role of circulating EVs in ischemic and hemorrhagic stroke has emerged as an intriguing area of research, as their levels have been found to correlate with clinical severity, the extent of the infarct area, and poor outcomes (contribution 3). In a study by Grossini et al., the authors combined the characterization of circulating EVs in subarachnoid hemorrhage (SAH) patients at various times with their cytopathic effect on two cell lines, endothelial cells and smooth muscle cells, which are considered to play a key role in the pathophysiology of SAH. The results of this study uncover the existence of an altered pattern of circulating EVs in SAH patients, detectable as early as 24 h post-SAH, suggesting that the characterization of EVs offers a promising non-invasive approach for screening SAH patients and has the potential to contribute to improved prognoses.

Bowers et al. (contribution 4) used a panel of transformed and non-transformed lung epithelial cell lines and human plasma to define the relationship between cellular and EV LINE-1 contents and to evaluate the expression of the oncogenic retrotransposon LINE-1 in healthy and diseased states. In their research paper, they present proof-of-principle that the expression of the oncogenic retrotransposon LINE-1 analytes in EVs serve as a “liquid biopsy” of LINE-1 tissue levels for molecular diagnostics in non-small-cell lung cancers (NSCLC). 

In the context of physiological processes, Meldolesi brings together studies that reveal that the neuronal EVs that are generated and active at pre-synaptic terminals appear different from those generated in other neuronal areas, and their activity could be part of the modulation of canonical neurotransmission signaling (contribution 5). In his opinion piece, the author suggests that future investigations could point to the role of synaptic EVs in pathologies related to circuits and trans-synaptic transmission to improve disease diagnosis and therapy.

In addition to these papers, this Special Issue deals with different aspects of the link between exosomes and disease and how to counteract them.

Abdulmalek et al. (contribution 6) offered a comprehensive review of the recent advances in stem cell-derived exosomes and their therapeutic applications in various diseases. In addition to providing a detailed description of their biogenesis, composition and signaling mechanism, their paper described various sources of exosomes indicating surface markers and therapeutic applications focusing on diabetes, polycystic ovary syndrome, gastrointestinal disorders, cardiovascular disease, cancer, plastic surgery, neurodegenerative disorders and lung disease. Interestingly, the authors described current clinical trials in which exosomes were used to showcase their promising therapeutic effects, although exosomes are known to have challenges and limitations, such as their maintenance, sterility and lack of standardized isolation protocols. Moreover, the outcomes of stem cell-derived exosomes are dependent upon their cargo, and, as concluded by the authors, by altering the cargo, a more individualized therapeutic effect can be achieved. In this context, Wang et al. (contribution 7) explored the therapeutic potential of exosomes loaded with paclitaxel for cancer therapy in vitro and in vivo. This work was impactful due to the use of a novel hybrid delivery system, exosome–liposome complexes, which significantly enhanced the stability and drug-loading capacity of paclitaxel. This system enhanced paclitaxel’s efficacy in colorectal tumor-bearing mice, offering interesting perspectives for effective cancer treatment strategies.

Manganelli et al. proposed an alternative raft target therapy for diseases in which the generation of exosomes is active. Using different methodologies for EV morphological analysis, they focused their attention on the autophagic process that triggers intracellular and extracellular vesicles (contribution 8).

Osaid et al. reviewed the role of exosomes in the physiological and pathological regulation of the blood–brain barrier (BBB) (contribution 9). Exosomes can cross the BBB and regulate its physiological functions when secreted by neurons, microglial cells and brain microvascular endothelial cells (BMEC); in particular, neural exosomes, containing miR-13, are able to reinforce the BBB’s integrity, driving the upregulation of tight junctions. In pathological conditions, the role of exosomes is controversial; they can promote or suppress disease pathogenesis, enhance tumor growth and disrupt the integrity of the BBB. On the contrary, in traumatic brain injury and stroke, they have therapeutic potential because of their ability to facilitate BBB regeneration. Moreover, they ameliorate BBB disruptions associated with Alzheimer’s and Parkinson’s diseases. However, the exact function of exosomes in degenerative processes warrants further investigation.

Crossing of the BBB by exosomes was addressed by Phelps et al. (contribution 10) in a research paper that compared the angiogenic capacity of EVs generated in stirred-tank or suspension bioreactors (SSBs) with EVs generated in traditional static T-flasks. The EVs produced in scalable, dynamic SSBs stimulated the angiogenesis of cerebral microvascular endothelial cells to a greater extent than EVs produced in standard static culture.

In conclusion, exosomes have specific advantages as potential biomarkers for disease prognosis and diagnosis, as drug carriers and for cell-free therapies. Despite the well-known limitations of exosomes, including variable cargo and routes of administration, potential side effects and a lack of standardized isolation protocols, all of these articles highlight their potential and innovative therapeutic applications and diagnostic approaches.

Considering the promise of EVs in the diagnosis and treatment of various conditions, challenges remain regarding their translation from the laboratory to the clinic. In this SI, we highlight the importance of correlating studies on physiological and pathological conditions to better address the prognostic and diagnostic value of EVs, and that by changing the load of EVs, it is possible to achieve a more individualized therapeutic effect from a tailored medicine perspective.
